# A scoping review of the questionnaires used for the assessment of the perception of undergraduate students of the learning environment in healthcare professions education programs

**DOI:** 10.1007/s10459-024-10319-1

**Published:** 2024-04-29

**Authors:** Banan Mukhalalati, Ola Yakti, Sara Elshami

**Affiliations:** Clinical Pharmacy and Practice Department, College of Pharmacy, QU Health, Qatar University, PO Box 2713, Doha, Qatar

**Keywords:** Learning environment, Health professions education programs, Perceptions, Questionnaires, Validity evidence

## Abstract

The learning environment (LE) includes social interactions, organizational culture, structures, and physical and virtual spaces that influence the learning experiences of students. Despite numerous studies exploring the perception of healthcare professional students (HCPS) of their LE, the validity evidence of the utilized questionnaires remains unclear. This scoping review aimed to identify questionnaires used to examine the perception of undergraduate HCPS of their LE and to assess their validity evidence. Five key concepts were used: (1) higher education; (2) questionnaire; (3) LE; (4) perception; and (5) health professions (HP). PubMed, ERIC, ProQuest, and Cochrane databases were searched for studies developing or adapting questionnaires to examine LE. This review employed the APERA standards of validity evidence and Beckman et al. (J Gen Intern Med 20:1159–1164, 2005) interpretation of these standards according to 5 categories: content, internal structure, response process, relation to other variables, and consequences. Out of 41 questionnaires included in this review, the analysis revealed a predominant emphasis on content and internal structure categories. However, less than 10% of the included questionnaires provided information in relation to other variables, consequences, and response process categories. Most of the identified questionnaires received extensive coverage in the fields of medicine and nursing, followed by dentistry. This review identified diverse questionnaires utilized for examining the perception of students of their LE across different HPs. Given the limited validity evidence for existing questionnaires, future research should prioritize the development and validation of psychometric measures. This will ultimately ensure sound and evidence-based quality improvement measures of the LE in HP education programs.

**Supplementary Information:**

The online version contains supplementary material available at 10.1007/s10459-024-10319-1.

## Introduction

Improving the learning experience of students in healthcare profession education programs (HPEPs) has been a demanding process in the healthcare professions education (HPE) (Carr et al., 2015). Indeed, HPEPs (e.g., pharmacy, medicine, nursing, and health sciences) are expected to prepare graduates with fundamental competencies, skills, and professional attributes and qualifications (Carr et al., 2015). Healthcare professional educators believe that the theoretical and clinical experiences that the students gain in their learning environment (LE) can significantly impact their attitudes, knowledge acquisition, skills development, and behaviors (Genn, 2001; Lizzio et al., 2002; Pimparyon, 2000). This is particularly important because the competencies of healthcare professionals influence patients' safety and ultimately health outcomes (Dunne et al., 2006).

The learning environment (LE) refers to the interactive combination of physical settings, educational resources, instructional approaches, and interpersonal dynamics that impact the learning journeys and experiences of students (Closs et al., 2022). According to Maudsley, a LE exists wherever and whenever students congregate, and it contains a variety of elements that support good instruction and serve as the curriculum's context (Maudsley, 2001). Hoidn (2016) argues that the LE demonstrates how various curricular components have an impact on students (Hoidn, 2016). Several studies have pointed out the important role that the LE has on the satisfaction and self-confidence of students (Al Ayed & Sheik, 2008; Lizzio et al., 2002; Wach et al., 2016; White, 2010). Therefore, accrediting bodies have increased their focus on the quality of the LE, highlighting that HPEPs are responsible for facilitating a positive LE, which supports the learning and professional development of students (Council, 1998; Education, 2009; Rusticus et al., 2020). Moreover, improving the LE has been recognized as a key standard in the World Federation for Medical Education (WFME) standards, which aim to ensure continuous quality improvement of medical education programs (Council, 1998).

According to (Genn, 2001), the crucial aspect lies in how students perceive their LE. The perception of students of their learning environment (LE) involves how learners perceive and make sense of the various elements, conditions, and factors that make up their educational surroundings (Genn, 2001). The perception can be enhanced by improving the motivation of students towards their learning and their interpersonal relationships, developing effective teaching strategies, and increasing the availability of infrastructure facilities (Genn, 2001). Additionally, enhancing the compliance of the higher education providers with cultural and international administrative standards within the physical environment is crucial for shaping a positive perception of the learning environment (Brown et al., 2011; Rawas & Yasmeen, 2019). Medical educators argue that the perception of students of their LE is one of the determinants of their academic and professional outcomes, therefore, assessing it is essential (Genn, 2001; Roff & McAleer, 2001).

Many educational institutes have investigated the perception of students of their LE regionally and internationally (Al Ayed & Sheik, 2008; Al-Hazimi et al., 2004a, 2004b; Lizzio et al., 2002; Rothman & Ayoade, 1970). In that regard, several studies indicated that the perception of students of their LE is affected by several factors, such as the gender of students and their academic achievement, as well as the curriculum content and the teaching styles (Cerón et al., 2016; Lokuhetty et al., 2010; Pimparyon, 2000). In a study conducted by a medical school that utilizes problem-based learning as a teaching strategy, first-year students exhibited neutral perception toward their LE, possibly due to their excitement upon entering the medical college; however, as they progressed in their study, they become more critical of the educational environment, indicating a shift in their perceptions over time (Nosair et al., 2015). Ahmed et al. (2018) argued that the perception of students of their LE and the factors that affect this perception should be assessed using reliable and comprehensive approaches (Ahmed et al., 2018). The approaches that have been used in the literature were quantitative (Rusticus et al., 2020) or qualitative (Britt et al., 2022; Fego et al., 2022) assessments. Quantitative assessment involves using validated and reliable questionnaires (RoffS et al., 1997; Rusticus et al., 2014, 2020), which should be ideally selected based on their comprehensiveness, quality, and validity evidence (Kishore et al., 2021). Furthermore, a key aspect to consider while assessing the comprehensiveness and robustness of a questionnaire is its theoretical foundation (Schönrock-Adema et al., 2012; Klein, 2016), because it reveals the key determinants of the measured outcome (Schönrock-Adema et al., 2012). Therefore, a review of the literature is required to identify the questionnaires used to assess the perception of students of their LE and to compare the quality of those questionnaires. The American Psychological and Educational Research Associations (APERA) have established standards for validity evidence, encompassing five key dimensions: (1) Content, (2) Response Process, (3) Internal Structure, (4) Relation to Other Variables, and (5) Consequences (Eignor, 2013). Content validity focuses on the development process and theoretical foundation of questionnaires. The response process centers on the analysis, accuracy, and thought processes related to respondents. Internal structure primarily addresses the reliability and factor analysis used to confirm the data structure of questionnaires. Relations to other variables examine the potential correlation between assessment scores and theoretically predicted outcomes or measures of the same construct. Consequences primarily describe the impact of assessment consequences on the validity of the score interpretation (Eignor, 2013).

The theoretical foundation/framework underpinning the questionnaire is a critical factor influencing its content validity, and hence its robustness (Beckman et al., 2005). Multiple theories and frameworks have been employed to ascertain the primary factors influencing the perceptions of students of their learning environment in the literature. Predominantly, experiential learning theory (Kolb, 1984), which emphasizes the central role that experience plays in the learning process, distinguishing this theory by its focus on experiential elements. Another common theory is the social theory (Bandura & Walters, 1977), which posits that individuals learn not only through direct experience but also by observing and imitating the behaviors of others. It also highlights the dynamic interaction between cognitive processes, environmental influences, and behavioral outcomes, and offers insights into how individuals acquire new behaviors through social interactions. Moos's framework (Moos, 1973, 1991) is the most commonly applied framework in the literature. Moos's renowned framework stands out for its emphasis on the interplay of environmental and interpersonal factors shaping individual experiences. Moos's conceptual model provides a nuanced perspective on the multifaceted influences that contribute to an individual's development and experiences, offering valuable insights into the realms of personal growth, social dynamics, and systemic adaptability (Moos, 1973, 1991).

Several systematic reviews were conducted to identify and compare the questionnaires that are used to examine the perception of healthcare professional students of their LE (Colbert-Getz et al., 2014; Hooven, 2014; Irby et al., 2021; Mansutti et al., 2017). These systematic reviews, however, were specific to one profession (Colbert-Getz et al., 2014; Hooven, 2014; Irby et al., 2021; Mansutti et al., 2017) or one setting (i.e., clinical versus preclinical). Only one systematic review, published in 2010, assessed the perception of students at a multidisciplinary level, including medicine, nursing and dentistry (Soemantri et al., 2010b). However, several newly developed questionnaires have been published after 2010 (Leighton, 2015; Rusticus et al., 2020; Shochet et al., 2015), including those emerged as a result of changes in the LE in the last years with the integration of artificial intelligence and virtual learning, and the development of educational and information technologies (Isba et al., 2020; Leighton, 2015; Rusticus et al., 2020; Shochet et al., 2015; Thibault, 2020). In that regard, no previous reviews included those newly developed questionnaires and examined the theoretical foundations of the developed questionnaires (Colbert-Getz et al., 2014; Hooven, 2014; Irby et al., 2021; Mansutti et al., 2017; Soemantri et al., 2010). Therefore, to overcome the potential gaps in the literature, this study aims to provide an up-to-date identification of questionnaires used to examine the perception of undergraduate healthcare professional students of their LE and to assess the quality of those identified questionnaires. The main objectives of this scoping review are to 1) categorize questionnaires used to assess the LE as perceived by undergraduate healthcare professional students based on development strategy, profession, and the setting; 2) identify the most commonly used questionnaires; 3) assess the validity evidence of the identified questionnaires; and 4) assess the theoretical foundation of the included questionnaires.

## Methods

### Protocol and Registration

This scoping review is compliant with the 2018 PRISMA statement for scoping reviews (PRISMA-ScR) (Tricco et al., 2018). The protocol for this scoping review was registered at RESEARCH REGISTRY and is available online at: [https://www.researchregistry. com/browse-the registry#registryofsystematicreviewsmetaanalyses/registryofsystematicreviewsmetaanalysesdetails/ 60070249970590001bd06f38/] with the number [reviewregistry1069].

## Eligibility criteria

This review aimed to identify articles that assess the perception of undergraduate healthcare professional students of their LE. While there is no universally established definition for healthcare professional educational programs or a standardized list of included educational professional programs, the researchers categorized these programs as educational programs associated with specific professions, namely, medicine, pharmacy, dentistry, nursing, and allied health. The term "allied health personnel" in PubMed's MeSH is utilized to define allied health, and relevant professions listed under this MeSH term. Studies were included if the following criteria were met: (1) used a questionnaire that was originally developed to assess LE in HPE; (2) focused on undergraduate students only, or both undergraduate and postgraduate students; (3) aimed to describe a questionnaire development, or to analyze the psychometric measures of a questionnaire, or to describe the utilization of a questionnaire; (4) published as research articles; and (5) published in peer-reviewed journals.

Studies were excluded if they (1) used a questionnaire that was not developed to assess LE in HPE; (2) focused on postgraduate students only; (3) did not describe the development, validity evidence, or the utilization of a questionnaire (i.e. studies that used only qualitative methods); (4) not research articles (e.g., theses and dissertations, conference papers, and abstracts); or (5) not published in peer-reviewed journals.

## Information sources

An electronic search was conducted in PubMed, ERIC, ProQuest, and Cochrane Library databases. The search was conducted between 1st July 2022 and 31st July 2022. Additional articles were identified from the reference lists of the identified articles and from other relevant reviews.

## Search strategy

The search strategy was developed by the research team (BM, OY, and SE), who are academics with expertise in pharmacy education and HPE research. The search strategy was revised by the Head of the Research and Instruction Section of the library at Qatar University, who has extensive expertise in health science, education, pharmacy, and medical databases.

Five main concepts were used “learning environment”, “healthcare professions”, “higher education”, “questionnaire”, and “perception”. Several keywords were identified for each concept (Appendix 1) and were matched to database-specific indexing terms. The identified concepts were combined using Boolean connectors (AND) and the keywords were combined using a Boolean connector (OR). The search results were then imported into EndNote version 9 and duplicates were identified and removed. The search was restricted to the English language, but no restriction was applied to the year of publication. A filter for peer-reviewed articles was used only when available. The detailed search strategy is demonstrated in Appendix 1.

## Selection of evidence sources

Two researchers (BM and OY) conducted the title/abstract screening for the identified articles. and excluded articles that are irrelevant to the research question based on the article title and abstract. Differences were resolved by a discussion with the third researcher (SE). The full-text screening was done by two investigators (OY and SE) who assessed the eligibility of the studies independently. Any disagreements were resolved by consensus via meetings and discussions. After the completion of the full-text screening, one researcher (OY) categorized the included questionnaires based on their utilization in the study into the following categories (originally developed questionnaires, adopted questionnaires, or adapted questionnaires). Studies that adopted a previously developed and validated questionnaire were not included in the data extraction of this scoping review, because they did not provide additional data about the development of the questionnaire or about the validity evidence of the questionnaire. However, the number of adoptions per questionnaire was recorded to address objective two of this review which is to identify the most commonly used questionnaires. In addition to the original development studies, adaptation studies that conducted psychometric measures testing, other than those done on the original development studies, were included in the data extraction.

## Data charting process and data items

Two researchers performed the data extraction independently using a data collection EXCEL sheet to tabulate data extracted from the included articles. The extracted data included the title of the manuscript, name of authors, year of publication, country where studies were conducted, aim and objectives of the research, study design, and study setting (i.e., clinical, preclinical, or both). Moreover, data related to the identified questionnaires were extracted, including the type of the questionnaire (i.e. new, adapted, or adopted), description of the domains and content, healthcare profession of which the research was conducted, and validity evidence of the questionnaire (including the use of theory or a theoretical framework in questionnaire development). Before the data extraction sheet was fully implemented, two investigators (SE and OY) piloted it using a sample of the articles from the review to determine its applicability, identify potential issues, and make the required changes. Piloting the data extraction sheet helped to improve the consistency and dependability of data extraction. Following successful piloting, the full data extraction was carried out using the data extraction sheet by the two investigators independently.

## Assessment of the psychometric properties of the included questionnaires

Studies that describe the development or assess the psychometric properties of questionnaires should ideally be based on high standards of methodological quality to be regarded as a legitimate and trustworthy instrument (Beckman et al., 2005). Data about the psychometric properties of the included questionnaires were collected, summarized, and assessed using the American Psychological and Education Research Associations (APERA) standards of validity evidence: (1) Content, (2) Response Process, (3) Internal Structure, (4) Relation to Other Variables, and (5) Consequences (Eignor, 2013), and using Beckman et al. (2005) interpretation of these standard categories (Beckman et al., 2005). Beckman et al. (2005) interpretation of these standard categories has been previously applied in various systematic reviews (Colbert-Getz et al., 2014; Fluit et al., 2010); including one systematic review that assessed the validity evidence of questionnaires that assess the perception of healthcare professional students of their LE(Colbert-Getz et al., 2014), aligning with the focus of this study. According to the assessment framework proposed by Beckman et al. (2005), each standard category was assigned a rating of N, 0, 1, or 2. The overall rating for each assessment tool was determined by calculating the total number of ratings corresponding to each standard category. However, it's important to note an overlap between "N" and "0″ ratings, where both can contribute to a zero-weight total score, despite their distinct interpretations. In response to this, the authors adopted a modified scoring system for the total sum score: "N" was treated as zero, "0″ as one, "1″ as two, and "2″ as three. Evaluating the theoretical basis of questionnaires was included in the total validity score, as part of APERA standards of validity evidence, under the ‘content’ category, where mentioning whether the questionnaire development was based on a theoretical basis and/or defining how this theoretical basis was applied/utilized would significantly change the score for the”content validity”. The definitions of Beckman et al. Table 1 summarizes the definitions of psychometric measures assessed by the Beckman et al. (2005) criteria and the interpretation of scores.

**Table 1:** Beckman et al. (2005) interpretation of APERA standards of validity evidence used to assess the quality of the included questionnaires

Test category	Rating	Interpretation
Content	N	No discussion of instrument content (includes simply listing items without justification)
	0	Discussion but no data
	1	Listing assessment themes with little or no reference to a theoretical basis, or a poorly defined process for creating and reviewing items
	2	A well-defined process for developing instrument content, including both an explicit theoretical/conceptual basis for instrument items and systematic item review by experts. Alternatively, reference to a prior study on an assessment instrument that meets these criteria
Response process	N	No discussion. Merely disclosing response rates or numbers of respondents does not constitute evidence
	0	Discussion but no data. Discussing the impact of response rate on assessment scores, or speculating on the thought processes of learners, does not constitute evidence
	1	Minimal data regarding thought processes and analysis of responses. Description (without data) of systems that reduce response error, such as computer-scored forms
	2	Multiple sources of supportive data, including critical examination of thought processes, analysis of responses for evidence of halo error or rater leniency, or data demonstrating low response error
Internal structure	N	No discussion
	0	Discussion but no data
	1	Factor analysis incompletely confirming anticipated data structure, or acceptable reliability with a single measure
	2	Factor analysis confirming anticipated data structure, or multiple measures of reliability. Variation in responses to specific items among subgroups (differential item functioning) can support or challenge internal structure depending on predictions
Relation to other variables	N	No discussion
	0	Discussion but no data
	1	Correlation of assessment scores to outcomes with minimal theoretical importance, or unanticipated score correlations
	2	Correlation (convergence) or no correlation (divergence) between assessment scores and theoretically predicted outcomes or measures of the same construct. Such evidence will usually be integral to the study design, and anticipated a priori
Consequences	N	No discussion. Speculation on potential applications of the assessment does not constitute evidence
	0	Discussion but no data. Simply discussing the consequences of assessment (e.g., data regarding usefulness or faculty approval) without linking this to validity does not constitute evidence
	1	Description of consequences of assessment that could conceivably impact the validity of score interpretations (although these impacts are not explicitly identified by the authors)
	2	Description of consequences of assessment that clearly impact on the validity of score interpretations, as supported by data and convincingly argued by the authors. Such evidence will usually be integral to the study design, and anticipated a priori

## Results

**Fig. 1:**
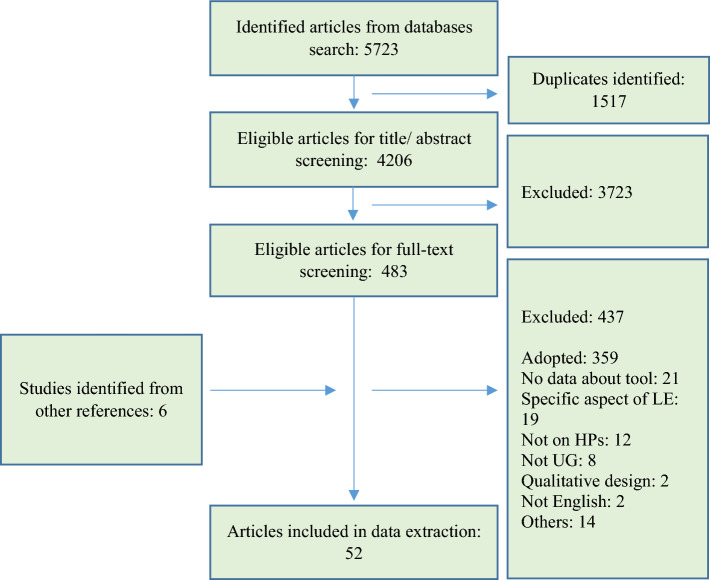
PRISMA flowchart of the article selection process

Out of 5723 articles retrieved from databases, 1517 articles were duplicates and were removed. After the title/abstract screening of 4206 articles, 3723 articles were irrelevant studies and excluded. This resulted in 483 articles eligible for full-text screening. After the full-text screening, 359 articles adopted previously developed questionnaires and were excluded because they did not provide any data about the psychometric properties of the adopted questionnaire. In addition, 72 articles were excluded for other reasons (i.e., were not conducted in HPE, did not include undergraduate students, assessed a specific aspect of the LE only, such as assessed LE of a specific course in the curriculum, did not provide data about the questionnaire development/ validation). Moreover, reviewing the reference lists of the eligible articles identified an additional six articles. This resulted in 52 articles eligible for data extraction; 41 articles were the original articles for the development of the questionnaires, and 11 articles were adaptation studies that tested one or more of the psychometric measures of the questionnaire. Figure 1 illustrates the PRISMA flowchart of the article selection process.

## Summary of the identified questionnaires

After the full-text screening, 41 questionnaires in the included articles were identified for data extraction. Table 2 provides a summary of the included questionnaires. The identified questionnaires in the included articles were divided into 3 categories, according to their development strategy. The first category included questionnaires developed based on a theoretical framework/theory, such as the Health Education Learning Environment Survey (HELES) (Rusticus et al., 2020), and the Manchester Clinical Placement Index (MCPI) (Dornan et al., 2012). The second category included adapted questionnaires, such as the Medical School Learning Environment Survey (MSLES) (Marshall, 1978) and the Dental Student Learning Environment Survey (DSLES) (Henzi et al., 2005). The third category included questionnaires developed through Delphi processes/ expert opinions, such as the Dundee Ready Educational Environment Measure (DREEM) questionnaire (Roff et al., 1997).

**Table 2:** Summary of the identified questionnaires

Profession	Questionnaire name, date, country	No. of Items	Setting	Type	Validity evidence (score)					Total validity score *	Theoretical basis
					Response process	Internal structure	Content	Relation to other variables	Consequences		
Nursing	CLES, (Saarikoski & Leino-Kilpi, 2002), Finland	27	Clinical	Original article		Internal consistency EFA	Yes				No
				Psychometric testing studies	Response rate Saarikoski et al., (2005)	PCA Saarikoski et al., (2005)		Compared to CLE scale (convergent validity) Saarikoski et al., (2005)			
				Score	(0)	(2)	(2)	(2)	(0)	11	
	CLEDI, (Hosoda, 2006), Japan	21	Clinical	Original study	Response rates	Internal consistency ICC EFA	Yes	Correlation with CLES (Hosoda, 2006) (Convergent validity)			Yes, experiential learning theory (Kolb, 1984) and epistemology of practice Schön, (1983)
				Score	(0)	(2)	(2)	(2)	(0)	11	
	CLES + T, Saarikoski et al., (2008), Finland	34	Clinical	Original article		Internal consistency EFA PCA		Compared to CALDs scale Compared to a general question for nurses satisfaction (Convergent validity)			Yes, framework cannot be determined
				Psychometric testing studies	Response rate Mikkonen et al., (2017)	Test re-test reliability Gustafsson et al., (2015)	Yes Johansson et al., (2010)	Discriminant validity Mikkonen et al., (2017)			
				Score	(0)	(2)	(2)	(2)	(0)	11	
	CLEI, (Chan, 2001; Chan, 2003), South Australia	35	Clinical	Original study	Response rate	Internal consistency	Yes	Convergent validity			Yes, Moos’s framework Moos, (1973)
				Psychometric testing studies		PCA Newton et al., (2010)					
				Score	(0)	(2)	(2)	(2)	(N)	10	
	Chuan et al., 2012 (Chuan & Barnett, 2012), Malasia	55	Clinical	Original article		Internal consistency PCA	Yes	Discriminant validity			Yes, name not mentioned
				Score	(N)	(2)	(2)	(2)	(N)	9	
	CLEI-19, (Salamonson et al., 2011), Australia	19	Clinical	Original article		Internal consistency PCA Inter-item analysis		Discriminant validity			No
				Score	(N)	(2)	(1)	(2)	(N)	8	
	CLE scale, (Dunn, 1995), United kingdom	23	Clinical	Original study		Internal consistency EFA CFA	Yes				Yes, Bloom’s Bloom, (1966)and Orton’s Orton, (1979)theories
				Score	(N)	(2)	(2)	(N)	(0)	7	
	CLECS, Leighton, (2015), United states	103	Clinical	Original article	Response rate	Internal consistency CFA	Yes				Yes. The National League for Nursing/Jeffries Simulation Framework (Jeffries, (2020)
				Psychometric testing studies		ICC Gu et al., (2018) EFA Gu et al., (2018) CFA (105)					
				Score	(0)	(2)	(2)	(N)	(N)	7	
	learning environment survey, Jessee et al., (2020), United states	142	Both	Original study	Response rate	Internal consistency	Yes				No
				Score	(1)	(1)	(2)	(N)	(N)	7	
	SECEE, Sand-Jecklin, (2009), United states	32	Clinical	Original article		Internal consistency EFA CFA	Yes				Yes, Cognitive Apprenticeship Collins and Kapur, (2006)
				Score	(N)	(2)	(2)	(N)	(N)	6	
	Modified CLES + T, D'Souza et al., (2015), Oman	57	Clinical	Original article	Response rate	Internal consistency	Yes				No
				Score	(0)	(1)	(2)	(N)	(N)	6	
	Oyira, (2016) Oyira et al., (2016), Nigeria	25	Pre-clinical	Original study		Split-half reliability	Yes				No
				Score	(N)	(1)	(2)	(N)	(N)	5	
	SNAP, Farrell and Coombes, (1994), United kingdom	9	Clinical	Original article	Yes		Yes				No
				Score	(1)	(N)	(1)	(N)	(N)	4	
	CLECS 2.0, Leighton et al., (2021), United states, Japan, Canada	29	Clinical	Original article			Face validity only		2 (		No
				Score	(N)	(N)	(1)	(N)	(N)	2	
Medicine	PLCS, Yılmaz et al., (2016), Turkey	52	Pre-clinical	Original article	Yes	Internal consistency The item-total correlation coefficients of EFA subscales	Yes	All the items significantly discriminated between low and high-performing students			No
				Score	(2)	(2)	(2)	(2)	(2)	15	
	JHLES, Shochet et al., (2015)	28	Both	Original article	Yes	Internal consistency EFA	Yes	Yes, compared to an overall JHLES score			Yes, social Bandura and Walters, (1977) and the experiential Kolb, (1984) learning theories
				Psychometric testing studies		Corrected item-total correlations Tackett et al., (2015)		Compared to DREEM Tackett et al., (2015)			
				Score	(2)	(2)	(2)	(2)	(0)	13	
	ECI, Krupat et al., (2017), United states	20	Pre-clinical	Original study	Yes	Internal consistency EFA CFA	Yes	ECI scores were different across six schools (Discriminant validity)			Yes, model of Carol Dweck Dweck, (2006)
				Score	(2)	(2)	(2)	(2)	(N)	12	
	CLEQS, Simpson et al., (2021), United States	10	Clinical	Original article		Internal consistency Item correlation	Yes				Yes, Gruppen framework Gruppen et al., (2019)
				Score	(1)	(1)	(2)	(2)	(0)	11	
	DREEM, Roff et al., (1997), United Kingdom	50	Pre-clinical	Original study		Internal consistency PCA	Yes	Compared to DMSQ (convergent validity)			No
				Psychometric testing studies	Response rate only Ahmad et al., (2015)	ICC Yusoff, (2012) EFA and CFA (Junaid Sarfraz et al., (2011)		Relationship between perception of LE and academic achievement/learning approach Jaffery and Kishwar, (2019) (Predictive validity)			
				Score	(0)	(2)	(2)	(2)	(N)	10	
	MCPI, Dornan et al., (2012), United Kingdom	8	Clinical	Original article	Response rate	Internal consistency Inter-rater reliability		–			Yes, CoP Wenger, (1999)
				Score	(0)	(2)	(1)	(2)	(N)	9	
	CLEQ, AlHaqwi et al., (2014), Saudi Arabia	40	Clinical	Original study	Response rate	Internal consistency PCA Factor analysis	Yes	Convergent validity Discriminated between different clinical LE (discriminant validity)			No
				Score	(0)	(2)	(1)	(2)	(N)	9	
	CMSS, Pololi et al., (2017), United States and Canada	51	Cannot determine	Original article	Response rate	Internal consistency Factor analysis		Discriminant validity			Yes, framework name not mentioned
				Score	(0)	(2)	(2)	(1)	(N)	9	
	HELES, (Rusticus et al., 2020), Canada	35	Both	Original study		Internal consistency EFA CFA	Yes	Compared to MSLES (convergent validity)			Yes, Moos’s framework Moos, (1973)
				Score	(N)	(2)	(2)	(2)	(N)	9	
	BLQ, Ballouk et al., (2022), Australia	19	Both	Original study		Internal consistency PCA EFA	Yes	Compared to MSLQ (Convergent validity)			No
				Score	(N)	(2)	(2)	(2)	(N)	9	
	UCEEM, Strand et al., (2013), Sweden	25	Clinical	Original article	Yes	Internal consistency EFA CFA Fouad et al., (2020)	Yes				Yes, contemporary workplace learning theory (Billett, 2002)
				Score	(0)	(2)	(2)	(0)	(N)	8	
	Abridged DREEM, (Jeyashree et al., 2018), India	12	Pre-clinical	Original article		Internal consistency Test–retest reliability CFA	Yes	Compared to DREEM-50 (convergent validity)			No
				Score	(N)	(2)	1	2	(N)	8	
	Modified MSLES, (Rosenbaum et al., 2007), United States	55	Pre-clinical	Original article		Reliability index Internal consistency Item- total correlation Item scale correlation		Discriminated between two different LEs (discriminant validity)			No
				Score	(N)	(2)	(N)	(2)	(N)	6	
	Pololi, Pololi et al., (2000), United States and Canada	31	Both	Original study		Internal consistency EFA	Yes				No
				Score	(N)	(2)	(1)	(N)	(N)	5	
	Shochet et al., (2013 Shochet et al., (2013), United States	55	Both	Original article	Response rate	Internal consistency	Yes				No
				Score	(N)	(1)	(2)	(N)	(N)	5	
	LEQ, Rothman & Ayoade, (1970), Canada		Cannot determine	Original article		Internal consistency	Face validity				No
				Score	(N)	(1)	(1)	(N)	(N)	4	
	MSEQ, Wakeford, (1981), United Kingdom	49	Cannot determine	Original article		Item analysis	Face validity				No
				Score	(N)	(1)	(1)	(N)	(N)	4	
	LEQ- shortened, Moore-West et al., (1986), United States	30	Pre-clinical	Original article		Internal consistency					No
				Score	(N)	(1)	(0)	(N)	(N)	3	
	Shortened MSLES, Rosenbaum et al., (2007), United States	17	Pre-clinical	Original article		Internal consistency Factor analysis					No
				Score	(N)	(2)	(N)	(N)	(N)	3	
	Johnson, 1978 (Johnson, 1978), United states		Pre-clinical	Original article		PCA					No
				Score	(N)	(1)	(N)	(N)	(N)	2	
	Parry et al., (2002) Parry et al., (2002), Umnited Kingdom	20	Clinical	Original article			Face validity only				No
				Score	(N)	(N)	(1)	(N)	(N)	2	
	MSEI, Hutchins, (1961), United States	180	Cannot determine	Original article							No
				Score	N	N	N	N	N	0	
Multidisciplinary	MSLES, 1978 Marshall, (1978), United States	50	Pre-clinical	Original study	Response rate	Internal consistency Test–retest reliability Split-half reliability CFA	Yes	Compared to DASS-21 And to JHLES (convergent validity)			No
				Psychometric testing studies		PCA Damiano et al., (2020)					
				Score	(0)	(2)	(2)	(2)	(N)	10	
	HEMLEM, Isba et al., (2020), United Kingdom	12	Clinical	Original study	Yes	Internal consistency EFA CFA	Yes				No
				Score	(2)	(2)	(2)	(N)	(N)	9	
	Jiboyewa & Umar, 2015 Jiboyewa et al., (2015), Nigeria	11	Pre-clinical	Original article	Response rate	Reliability index: 0.68	Yes				Yes, open system theory and transformational leadership theory
				Score	(0)	(1)	(2)	(N)	(N)	6	
Dentistry	DSLES, Henzi et al., (2005), North America	55	Pre-clinical	Original article		Internal consistency	Yes				No
				Score	(0)	(1)	(1)	(N)	(N)	5	
	DCLEI, Kossioni et al., (2014), Greese	42	Both	Original study	Yes	EFA PCA	Yes	Discriminant validity			No
				Score	(2)	(2)	(2)	(2)	(N)	12	

^*^Total validity score is calculated as follows: "N" was treated as zero, "0″ as one, "1″ as two, and "2″ as three.”

Abbreviations: EFA: Exploratory Factor Analysis; CFA: Confirmatory Factor Analysis; PCA: Principle Component Analysis; ICC: Intraclass Correlation Coefficient; DMSQ: Cop: Community Of Practice; BLQ: Blended Learning Questionnaire; MSLQ: The Motivated Strategies For Learning Questionnaire; CLE: Clinical Learning Environment; DCLEI: Dental Clinical Learning Environment Instrument; ECI: The Educational Climate Inventory; DASS-21: The Depression, Anxiety And Stress Scale—21; CAs: The Culturally And Linguistically Diversity scale; UCEEM: Undergraduate Clinical Education Environment Measure; MSEQ: Medical School Environment Questionnaire; MSEI: Medical School Environment Inventory; SECEE: The Student Evaluation of Clinical Education Environment; SNAP: Student Nurse Appraisal of Placement; CMSS: C-Change Medical Student Survey; LEQ: Learning Environment Questionnaire

**Fig. 2:**
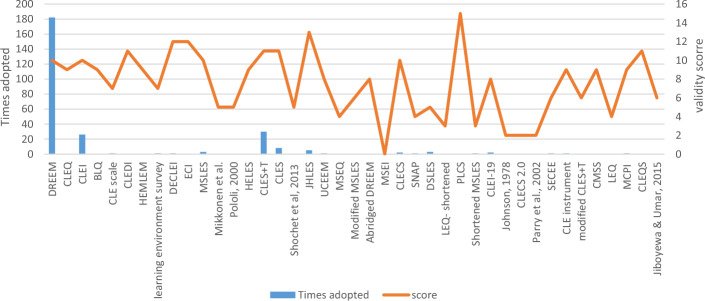
Total validity evidence scores versus the number of adoptions of the included questionnaires

The majority of the identified questionnaires in the included articles were originally developed for one profession and, hence, were suitable to examine aspects specific to the context of that profession. For example, a total of 22 questionnaires out of the 41 identified questionnaires were specific to the medical profession. DREEM was originally developed for the medical profession, was the most adopted questionnaire across the medical profession and other HPs (Fig. 2), and it was translated into more than 5 languages (Al-Hazimi et al., 2004a, 2004b; Andalib et al., 2015; Demiroren et al., 2008; Dimoliatis et al., 2010; Miles et al., 2012). Fourteen questionnaires were developed specifically for the nursing profession, with CLES + T being the most widely adopted and translated into multiple languages (Johansson et al., 2010; Tomietto et al., 2012; Vizcaya-Moreno et al., 2015). For the dentistry profession, only two questionnaires were identified: DECLEI and DSLES, where DSLES was adopted more in subsequent dentistry profession studies than DECLEI. Only a few questionnaires were originally developed to evaluate the perception of multidisciplinary students of their LE. However, some questionnaires that were originally developed for a specific profession were utilized to evaluate the perception of students of their LE in other professions. For example, although (e.g., DREEM) was initially administered among medical students, it was also pilot-tested in the nursing profession, in the original development study (Roff et al., 1997) and then was adopted in the dental (Ali et al., 2012), health-sciences (Sunkad et al., 2015), and nursing professions (Abusaad et al., 2015). Regarding the setting for which the identified questionnaire in the included articles was developed, some questionnaires were developed to evaluate the perception of students of their LE in the clinical setting (e.g., the Clinical Learning Environment Inventory (CLEI) and MCPI), while others were used to evaluate the perception of students of their LE in both non-clinical and clinical settings (e.g., DREEM). Table 4 summarizes the identified questionnaires based on the settings of the LE and the profession.

## The psychometric properties of the identified questionnaires

Evaluation of the psychometric properties of the questionnaires in the included articles was conducted using the five standard categories of APERA standards of validity evidence: content validity, response process, internal structure, relation to other variables, and consequences (Eignor, 2013), and using Beckman et al. (2005) interpretation of these standard categories (Beckman et al., 2005). Content validity and internal structure categories were reported and assessed in most of the questionnaires, while the response process and relation to other variables measures were reported and assessed in a smaller number of studies. Only one questionnaire; the Preclinical Learning Climate Scale (PLCS) provided data on the five psychometric measures (Yılmaz et al., 2016). Furthermore, the PLCS has the highest Beckman et al. (2005) total validity score among other questionnaires, followed by the Johns Hopkins Learning Environment Scale (JHLES). Evaluating the validity evidence of the questionnaires in the included articles based on the profession for which they were originally developed demonstrated that Clinical Learning Environment Quick Survey (CLEQS) (Simpson et al., 2021), followed by DREEM (Roff et al., 1997) scored the highest among the questionnaires developed for the medical profession. Whereas the Clinical Learning Environment, Supervision and Nurse Teacher (CLES + T) Scale (Saarikoski et al., 2008), the Clinical Learning Environment and Supervision (CLES) instrument (Saarikoski & Leino-Kilpi, 2002), and the Clinical Learning Environment Diagnostic Inventory (CLEDI) (Hosoda, 2006) scored the highest among questionnaires developed for the nursing profession, followed by the Clinical Learning Environment Comparison Survey (CLECS) (Leighton, 2015). Evaluating the validity evidence of the questionnaires developed for the dentistry profession indicated that DECLEI had a higher validity evidence score than DSLES. Finally, the MSLES (Marshall, 1978) followed by the Healthcare Education Micro-Learning Environment Measure (HEMLEM) (Isba et al., 2020) scored the highest among questionnaires developed for multidisciplinary. Table 3 provides a summary of the validity evidence of the identified questionnaires in the included articles.

**Table 3:** Summary of the validity evidence and total validity score of the identified questionnaires

Profession	Questionnaire name	Validity evidence (score)					Total validity score*
		Response process	Internal structure	Content	Relation to other variables	Consequences	
Nursing	CLES, 2002 (Saarikoski & Leino-Kilpi, 2002)	(0)	(2)	(2)	(2)	(0)	11
	CLEDI, 2006 (Hosoda, 2006)	(0)	(2)	(2)	(2)	(0)	11
	CLES + T, 2008 Saarikoski et al., (2008)	(0)	(2)	(2)	(2)	(0)	11
	CLEI, 2001, 2003 (Chan, 2001; Chan, 2003)	(0)	(2)	(2)	(2)	(N)	10
	Chuan et al., 2012 Chuan and Barnett, (2012)	(N)	(2)	(2)	(2)	(N)	9
	CLEI-19, 2011 (Salamonson et al., 2011)	(N)	(2)	(1)	(2)	(N)	8
	CLE scale, 1995 (Dunn, 1995)	(N)	(2)	(2)	(N)	(0)	7
	CLECS, 2015 Leighton, (2015)	(0)	(2)	(2)	(N)	(N)	7
	Learning environment survey, 2020 Jessee et al., (2020)	(1)	(1)	(2)	(N)	(N)	7
	SECEE, 2009 Sand-Jecklin, (2009)	(N)	(2)	(2)	(N)	(N)	6
	modified CLES + T, 2015 D'Souza et al., (2015)	(0)	(1)	(2)	(N)	(N)	6
	Oyira, 2016 Oyira et al., (2016)	(N)	(1)	(2)	(N)	(N)	5
	SNAP, 1994 Farrell and Coombes, (1994)	(1)	(N)	(1)	(N)	(N)	4
	CLECS 2.0, 2021 Leighton et al., (2021)	(N)	(N)	(1)	(N)	(N)	2
Medicine	PLCS, 2016 Yılmaz et al., (2016)	(2)	(2)	(2)	(2)	(2)	15
	JHLES, 2015 Shochet et al., (2015)	(2)	(2)	(2)	(2)	(0)	13
	ECI, 2017 (Krupat et al., 2017)	(2)	(2)	(2)	(2)	(N)	12
	CLEQS, 2021 Simpson et al., (2021)	(1)	(1)	(2)	(2)	(0)	11
	DREEM, 1997 Roff et al., (1997)	(0)	(2)	(2)	(2)	(N)	10
	MCPI, 2012 Dornan et al., (2012)	(0)	(2)	(1)	(2)	(N)	9
	CLEQ, 2014 AlHaqwi et al., (2014)	(0)	(2)	(1)	(2)	(N)	9
	CMSS, 2017 (Pololi et al., 2017)	(0)	(2)	(2)	(1)	(N)	9
	HELES, 2020 Rusticus et al., (2020)	(N)	(2)	(2)	(2)	(N)	9
	BLQ, 2022 Ballouk et al., (2022)	(N)	(2)	(2)	(2)	(N)	9
	UCEEM, 2013 (Strand et al., 2013)	(0)	(2)	(2)	(0)	(N)	8
	Abridged DREEM, 2018 Jeyashree et al., (2018)	(N)	(2)	(1)	(2)	(N)	8
	Modified MSLES, 2007 Rosenbaum et al., (2007)	(N)	(2)	(N)	(2)	(N)	6
	Pololi, 2000 (Pololi et al., 2000)	(N)	(2)	(1)	(N)	(N)	5
	Shochet et al., 2013 Shochet et al., (2013)	(N)	(1)	(2)	(N)	(N)	5
	LEQ, 1970 Rothman & Ayoade, (1970)	(N)	(1)	(1)	(N)	(N)	4
	MSEQ, 1981 Wakeford, (1981)	(N)	(1)	(1)	(N)	(N)	4
	LEQ- shortened, 1986 (Moore-West et al., 1986)	(N)	(1)	(0)	(N)	(N)	3
	Shortened MSLES, 2007 Rosenbaum et al., (2007)	(N)	(2)	(N)	(N)	(N)	3
	Johnson, (1978) Johnson, (1978)	(N)	(1)	(N)	(N)	(N)	2
	Parry et al., (2002) Parry et al., (2002)	(N)	(N)	(1)	(N)	(N)	2
	MSEI, 1961 Hutchins, (1961)	(N)	(N)	(N)	(N)	(N)	0
Multidisciplinary	MSLES, 1978 Marshall, (1978)	(0)	(2)	(2)	(2)	(N)	10
	HEMLEM, 2020 (Isba et al., 2020)	(2)	(2)	(2)	(N)	(N)	9
	Jiboyewa & Umar, 2015 Jiboyewa et al., (2015)	(0)	(1)	(2)	(N)	(N)	6
Dentistry	DCLEI, 2014 Kossioni et al., (2014)	(2)	(2)	(2)	(2)	(N)	12
	DSLES, 2005 Henzi et al., (2005)	(0)	(1)	(1)	(N)	(N)	5

^*^ Total validity score is calculated as follows: "N" was treated as zero, "0″ as one, "1″ as two, and "2″ as three.”

Abbreviations: EFA: Exploratory Factor Analysis; CFA: Confirmatory Factor Analysis; PCA: Principle Component Analysis; ICC: Intraclass Correlation Coefficient; DMSQ: Cop: Community Of Practice; BLQ: Blended Learning Questionnaire; MSLQ: The Motivated Strategies For Learning Questionnaire; CLE: Clinical Learning Environment; DCLEI: Dental Clinical Learning Environment Instrument; ECI: The Educational Climate Inventory; DASS-21: The Depression, Anxiety And Stress Scale—21; CAs: The Culturally And Linguistically Diversity scale; UCEEM: Undergraduate Clinical Education Environment Measure; MSEQ: Medical School Environment Questionnaire; MSEI: Medical School Environment Inventory; SECEE: The Student Evaluation of Clinical Education Environment; SNAP: Student Nurse Appraisal of Placement; CMSS: C-Change Medical Student Survey; LEQ: Learning Environment Questionnaire

Figure 2 demonstrates the relationship between the validity evidence score of each questionnaire and the frequency of subsequent use (adoption) of the questionnaire. The adoption of the majority of the identified questionnaires in subsequent studies was limited, with DREEM, CLES + T, CLEI, CLES, JHLES, and MSLES being the most frequently adopted questionnaires. Notably, although PLCS had the highest validity evidence score among all identified questionnaires, it was not adopted in subsequent studies. While DREEM was the most frequently adopted questionnaire, it ranked ninth in the validity evidence score.

## Framework use

Less than half of the identified questionnaires in the included articles (n = 15/41) were developed based on a theory or a theoretical framework, such as the JHLES, HELES, and CLEI questionnaires. The most commonly used theory in the development of the questionnaires was the experiential learning theory which emphasizes learning through experience and reflection (Kolb, 1984). Additionally, Moos's framework, known for its focus on environmental and interpersonal factors influencing individuals, was the most commonly used theoretical framework (Moos, 1973). The theoretical frameworks and theories utilized are summarized in Table 2.

## Discussion

This scoping review aims to identify questionnaires used to examine the perception of undergraduate healthcare professional students of their LE and to assess the validity evidence of those identified questionnaires. This review resulted in identifying original or adapted questionnaires used to assess the perception of undergraduate healthcare professional students of their LE and in providing an assessment of their validity evidence. This review shed light on the most frequently reported psychometric properties for developing and validating questionnaires that were used to assess the perception of healthcare professional students of their LE in HPEPs, as well as on the trends of adopting LE questionnaires across different HPEPs (Table 4).

**Table 4:** Summary of the identified questionnaires

Healthcare professions	Clinical	Pre-clinical	Both environments	Total*
Nursing	12	1	1	14
Medicine	4	8	5	13
Dentistry	1	1	1	3
Multidisciplinary	1	2	–	3
Total	18	12	7	37

^*^Clinical/pre-clinical cannot be determined for status for four questionnaires

The findings of this review suggested that DREEM was the most commonly used questionnaire for examining LE in medical profession and across different professions, and was widely adopted in various countries and cultures worldwide (Dimoliatis et al., 2010; Soemantri et al., 2010). This finding aligns with the results of Colbert-Getz et al.’s (2014) systematic review (Colbert-Getz et al., 2014), which argued that DREEM, initially developed by international students in Dundee University Medical School, achieved widespread usage as these students implemented it in their respective institutions (Colbert-Getz et al., 2014). Moreover, Colbert-Getz et al. (2014) claimed that researchers usually choose DREEM, because it is one of the oldest and most widely adopted questionnaires (Colbert-Getz et al., 2014). This prompts researchers to adopt DREEM to facilitate comparisons of their findings on students' perceptions of their learning environment with other institutions that have employed the same questionnaire before (Miles et al., 2012). It is worth noting that despite the length of DREEM questionnaire (50 items), the questions are generally easy to comprehend, which could have potentially facilitated its popularity and spread.

This review demonstrated that the majority of the questionnaires have limited validity evidence, where ‘content validity’ and ‘internal structure’ were the most reported validity evidence categories of APERA standards. Furthermore, the majority of the questionnaires did not have a thorough assessment of the ‘response process’, ‘relation to other variables’, and the ‘consequences’ categories. This finding is consistent with Colbert-Getz et al.’s (2014) systematic review of studies in medical education, which utilized APERA standards and Beckman et al. (2005) interpretation (Colbert-Getz et al., 2014). Moreover, this finding is in line with Mansutti et al.’s (2017) systematic review of studies in nursing education, which utilized the consensus-based standards for the selection of health measurement instruments (COSMIN) tool (Mansutti et al., 2017) to evaluate the methodological quality of the psychometric properties of instruments developed to assess the clinical LE in the nursing education (Mansutti et al., 2017). The COSMIN tool facilitates a more comprehensive assessment of both psychometric properties and research methods, organized into distinct dimensions labeled in alignment with the property being evaluated. This includes internal consistency, reliability, measurement error, content validity (including face validity), structural validity, hypotheses testing (including convergent validity), criterion validity, cross-cultural, responsiveness, interpretability, and generalisability of the findings. Mansutti et al.’s (2017) systematic review revealed that concept and construct validity were inadequately addressed and infrequently evaluated by the nursing student population. Whereas, some properties, such as reliability, measurement error, and criterion validity, were rarely considered (Mansutti et al., 2017). Limited validity evidence of the developed questionnaires continues to be a challenge in the health literature (Bai et al., 2008; Hirani et al., 2013). This challenge was explained by Boateng et al. (2018) who argued that the process of instrument development and validation is complex and requires knowledge and skills in sophisticated statistical analysis methods (Boateng et al., 2018). However, several graduate programs in behavioral and health sciences do not adequately account for those statistical analysis methods in training and educating their students (Boateng et al., 2018).

In this review, PLCS demonstrated the highest validity evidence on the five APERA standards categories among all questionnaires (Yılmaz et al., 2016). PLCS was developed in 2016, and hence it was not identified in Soemantri et al.’s (2010) (Soemantri et al., 2010) and in Colbert-Getz et al.’s (2014) (Colbert-Getz et al., 2014) systematic reviews. In 2010, Soemantri et al. systematic review utilized three types of validity assessment (i.e., content, criterion-related, and construct) and argued that DREEM is the best questionnaire for examining the perception of undergraduate medical students of their LE (Soemantri et al., 2010). However, Soemantri et al.’s approach to evaluating the content, criterion-related, and construct validities did not include essential psychometric properties, such as response process, internal structure, and consequences. Consequently, DREEM received a higher score in Soemantri et al.’s review compared to the current review, where a relatively lower validity evidence score was assigned, adhering to APERA standards and Beckman et al. (2005) interpretation. In Colbert-Getz et al.’s (2014) systematic review (Colbert-Getz et al., 2014), Pololi and Price's (2000) questionnaire (Pololi & Price, 2000) received the highest validity evidence score using APERA standards; however, Colbert-Getz et al. did not take the category ‘consequences’ into consideration (Colbert-Getz et al., 2014). Thus, Pololi and Price's (2000) questionnaire obtained a lower validity evidence score in the current review, aligning with APERA standards and Beckman et al. (2005) interpretation. CLES + T received the highest validity evidence among questionnaires developed for the nursing profession in the current review as well as in Mansutti et al.’s (2017) systematic review (Mansutti et al., 2017), which utilized the COSMIN tool for evaluating research methods and psychometric properties of instruments designed to assess the clinical LE in the nursing education (Mansutti et al., 2017). The consistency between Mansutti et al.’s (2017) systematic review and the current review potentially suggests the applicability of the use of APERA standards and Beckman et al. (2005) interpretation for the psychometric testing assessment of questionnaires in HPE.

The utilization of theory or theoretical framework in questionnaire development ensures that the research findings are theory-driven, which enhances their robustness and rigor (Schönrock-Adema, 2012; Stewart & Susan Klein, 2016). This review revealed that less than fifty percent of the included articles, (17/41), utilized a theory or a theoretical framework in the questionnaire development process. This finding was supported by other studies that indicated that the development of questionaries for examining the perception of healthcare professional students of their LE usually lacks solid grounding on theoretical frameworks. This was justified by Schnrock-Adema et al. by the lack of consensus about the most suitable framework to assess the LE (Schönrock-Adema, 2012). Remarkably, the development of both DREEM, which is the most commonly used questionnaire and PLCS, which is the most valid questionnaire was not grounded on a theoretical basis. The findings of this scoping review suggest that the two most frequently utilized theories and theoretical frameworks in the development of the questionnaires in the included articles were Kolb’s (1984) experiential learning theory (Kolb, 1984), and Moos’s (1973, 1991) learning environment framework (Moos, 1973, 1991), respectively. According to Kolb (1984)’s experiential learning theory, learning and knowledge development takes place through engagement with the real-world environment (Abdulwahed, 2010), which further highlights that the LE plays an indispensable role in the learning process and significantly influences the learning experience, performance, and learning outcome of students (Kolb, 1984). Moos’s (1973, 1991) learning environment framework provided an integrated system approach, which analyzes the LE and the LE effect on learning experiences and outcomes holistically (Moos, 1973, 1991). Moos’s framework is composed of three elements: ‘personal development’, ‘relationships’, and ‘system maintenance and change’ (Insel & Moos, 1974; Moos, 1973, 1991). The ‘personal development’ element comprises the opportunities within an environment and the capacity for personal growth and self-esteem improvement. The ‘relationship’ element involves the extent to which individuals deal with and support each other in an environment. The ‘system maintenance and change’ element represents the environmental physical dimension, in terms of clarity and transparency to change within an institutional structural setting (Insel & Moos, 1974; Moos, 1973, 1991). In the current review, Kolb’s (1984) experiential learning theory and Moos’s (1973, 1991) learning environment framework were utilized for developing questionnaires that were intended to be used in clinical, experiential learning settings, such as HEMLEM (Isba et al., 2020) and CLEI (Chan, 2001; Chan, 2003), as well as in those that were intended to be used in both, clinical and academic settings such as JHLES (Shochet et al., 2015) and HELES (Rusticus et al., 2020).

## Limitations and strengths

This is the first review that provides a comprehensive and critical assessment of questionnaires that are used to assess the perception of undergraduate students of their LE in HPEPs, with no restriction to profession, or setting. Moreover, this review is unique in indicating whether a theory or a theoretical framework was utilized in the development of the questionnaire.

Nevertheless, a few limitations should be recognized when interpreting the findings of this review. Although the use of the APERA standards of validity evidence provided a valuable assessment of the quality of the included questionnaires, other reviews have used more detailed and comprehensive criteria (Mansutti et al., 2017), such as the COSMIN tool. Using the COSMIN tool in this review was not practical because of its cognitively demanding nature. Another point that should be taken into consideration when interpreting the findings of this review is the total validity score. Adopting the APERA lens for validity assessment and Beckman et al. (2005) interpretation assumes equal weight for all five evidence sources and disregards potential differences in their significance, which depends on the specific use context of the assessment. A more flexible and nuanced approach to validity arguments is provided by Kane's framework, which enables prioritization according to the assessment's purpose and inferences as well as a personalized focus on pertinent data (Cook et al., 2015; Kane, 2006, 2013). Kane's framework would be an invaluable resource for educators seeking a more thorough and context-sensitive knowledge of assessment validity. Again, the cognitively demanding nature of Kane's framework rendered its application impractical for this review. An additional limitation is that this review did not report the interrater reliability for scoring the sources of validity evidence for each questionnaire. This could have been beneficial in providing valuable insights into the consistency of judgments among reviewers and understanding the potential limitations of using the adopted methodology. Nevertheless, an attempt was made during the data extraction stage to enhance the interrater reliability of the validity evidence assessment of the questionnaires by piloting the data extraction sheet on a sample of the included articles. It is worth mentioning, however, that challenges in consistently measuring and evaluating evidence for specific APERA categories of validity evidence may result from the scarcity of reported evidence related to those categories (Beckman et al., 2005). Furthermore, the search in this review was limited to three databases, possibly leading to the exclusion of significant articles exclusive to other databases. Nonetheless, a comprehensive review of the reference lists in the included articles was conducted to identify relevant studies. Finally, restricting the search to English-language publications has potentially resulted in excluding valuable research articles published in other languages, which could affect the generalizability and comprehensiveness of the findings of this scoping review.

## Conclusions

This scoping review provided an overview of the available questionnaires in the HPE literature to assess the perception of undergraduate students of their LE. The review also provided a summary of the validity evidence and theoretical basis of the identified questionnaires. A total of 41 questionnaires were identified in the included articles for different HPEPs. The results suggested that DREEM, CLES + T, and CLEI were the most commonly used questionnaires, while PLCS followed by JHLES had the highest total validity evidence score, using the APERA standards of validity evidence and Beckman et al. (2005) interpretation of these standard categories. Moreover, this review demonstrated that only a few questionnaires in the included articles were designed using a theoretical foundation. Furthermore, the findings of this research suggested that the newly developed questionnaires that are theoretically driven had well-established validity evidence. Therefore, a culture of developing and validating questionnaires according to high standards and best practices needs to be adopted and reinforced by healthcare professional educators to ensure the rigor of studies conducted to improve the quality of the LE. Furthermore, the investigators of the current review strongly advocate for a shift from adopting questionnaires based on the wide spread of use to that based on validity and reliability evidence, as well as to contribute to establishing the psychometric measures of the newly developed ones. Finally, this review did not reveal any questionnaire that was specifically developed to assess the perception of students of their LE in some of the major HPEPs such as pharmacy or biomedical sciences. Consequently, healthcare professional educators and scholars are encouraged to examine the common aspects of the LE within their respective health professions, and ultimately plan to investigate those common aspects across various HPEPs in order to understand how they influence the perception of students of their learning experiences and outcome.

### Supplementary Information

Below is the link to the electronic supplementary material.

Supplementary file1 (DOCX 41 KB)
