# Supplementary material for: A scoping review of the questionnaires used for the assessment of the perception of undergraduate students of the learning environment in healthcare professions education programs
Source: Adv Health Sci Educ Theory Pract. 2024 Apr 29;29(4):1501–38. doi: 10.1007/s10459-024-10319-1 (PMC11369005; doi:10.1007/s10459-024-10319-1)
Supplement: Supplementary file 1 — Supplementary file1 (DOCX 41 KB) [file 10459_2024_10319_MOESM1_ESM.docx]

Appendix 1. Search strategies for all searched databases

| **Database** | **Search strategy** | **Filters** | **Results** |
| --- | --- | --- | --- |
| **Pubmed** | ((((((((((((((((((((((((("academic environment"[Title/Abstract]) OR ("academic climate"[Title/Abstract])) OR ("learning environment"[Title/Abstract])) OR ("learning climate"[Title/Abstract])) OR ("classroom environment"[Title/Abstract])) OR ("classroom climate"[Title/Abstract])) OR ("education environment"[Title/Abstract])) OR ("education climate"[Title/Abstract])) OR ("education setting"[Title/Abstract])) OR ("educational environment"[Title/Abstract])) OR ("educational climate"[Title/Abstract])) OR ("educational setting"[Title/Abstract])) OR ("learning setting"[Title/Abstract])) OR ("academic setting"[Title/Abstract])) OR ("academic environments"[Title/Abstract])) OR ("academic climates"[Title/Abstract])) OR ("learning environments"[Title/Abstract])) OR ("learning climates"[Title/Abstract])) OR ("classroom environments"[Title/Abstract])) OR ("classroom climates"[Title/Abstract])) OR ("education settings"[Title/Abstract])) OR ("educational environments"[Title/Abstract])) OR ("educational climates"[Title/Abstract])) OR ("educational settings"[Title/Abstract])) OR ("learning settings"[Title/Abstract])) OR ("academic settings"[Title/Abstract]) AND (((((((((((((((((((((((((((((((("Health professions"[Title/Abstract])) OR ("Health profession"[Title/Abstract])) OR ("Health professional"[Title/Abstract])) OR ("Health professionals"[Title/Abstract])) OR ("Healthcare professions"[Title/Abstract])) OR ("Healthcare profession"[Title/Abstract])) OR (" Healthcare professional"[Title/Abstract])) OR ("Healthcare professionals"[Title/Abstract])) OR ("Health care professions"[Title/Abstract])) OR ("Health care profession"[Title/Abstract])) OR ("Health care professional"[Title/Abstract])) OR ("Health care professionals"[Title/Abstract])) OR ("Allied health"[Title/Abstract])) OR ("Health science"[Title/Abstract])) OR ("Health sciences"[Title/Abstract])) OR (Biomed*[Title/Abstract])) OR (dent*[Title/Abstract])) OR (nurs*[Title/Abstract])) OR (nutrition*[Title/Abstract])) OR (diet*[Title/Abstract])) OR (physiotherap*[Title/Abstract])) OR (physio*therap*[Title/Abstract])) OR (occupational therap*[Title/Abstract])) OR (occupation therap*[Title/Abstract])) OR (rad*therap*[Title/Abstract])) OR (radiotherap*[Title/Abstract])) OR (radiation therap*[Title/Abstract])) OR ((physical therap*[Title/Abstract])) OR (veterinar*[Title/Abstract])) OR (medical[Title/Abstract])) OR (medicine[Title/Abstract])) OR (health personnel[MeSH Terms]) OR (pharmac*[Title/Abstract]) AND (((((((((((school*[Title/Abstract]) OR (college*[Title/Abstract])) OR (universit*[Title/Abstract])) OR (education[MeSH Terms])) OR (faculty[Title/Abstract]) ) OR ("learning institute"[Title/Abstract])) OR ("learning institutes"[Title/Abstract])) OR ("learning institution"[Title/Abstract])) OR ("learning institutions"[Title/Abstract]) OR ("education institute"[Title/Abstract])) OR ("education institutes"[Title/Abstract])) OR ("education institution"[Title/Abstract])) OR ("education institutions"[Title/Abstract]) OR ("educational institute"[Title/Abstract]) OR ("educational institutes"[Title/Abstract]) OR ("educational institution"[Title/Abstract]) OR ("educational institutions"[Title/Abstract]) OR ("academic institute"[Title/Abstract]) OR ("academic institutes"[Title/Abstract]) OR ("academic institution"[Title/Abstract]) OR ("academic institutions"[Title/Abstract]) AND ((((((tool*[Title/Abstract]) OR (instrument*[Title/Abstract])) OR (survey*[Title/Abstract])) OR (quantitative*[Title/Abstract])) OR (questionnaire*[Title/Abstract])) OR (scale*[Title/Abstract])) OR (inventor*[Title/Abstract]) AND (((((((((((((((opinion*[Title/Abstract])) OR (attitude*[Title/Abstract])) OR (view[Title/Abstract])) OR (perspective*[Title/Abstract])) OR (perceive*[Title/Abstract])) OR (behaviour*[Title/Abstract])) OR (percept*[Title/Abstract]) ) OR (measur*[Title/Abstract])) OR (assess*[Title/Abstract]))) OR (evaluat*[Title/Abstract]))) OR (views[Title/Abstract])) OR (viewpoint[Title/Abstract])) OR (viewpoints[Title/Abstract]) | English language  Since 1940 | 2,942 |
| **ProQuest** | (TI("academic environment" OR "academic climate" OR "learning environment" OR "learning climate" OR "classroom environment" OR "classroom climate" OR "education environment" OR "education climate" OR "education setting" OR "educational environment" OR "educational climate" OR "educational setting" OR "learning setting" OR "academic setting" OR "academic environments" OR "academic climates" OR "learning environments" OR "learning climates" OR "classroom environments" OR "classroom climates" OR "education settings" OR "educational environments" OR "educational climates" OR "educational settings" OR "learning settings" OR "academic settings") OR AB("academic environment" OR "academic climate" OR "learning environment" OR "learning climate" OR "classroom environment" OR "classroom climate" OR "education environment" OR "education climate" OR "education setting" OR "educational environment" OR "educational climate" OR "educational setting" OR "learning setting" OR "academic setting" OR "academic environments" OR "academic climates" OR "learning environments" OR "learning climates" OR "classroom environments" OR "classroom climates" OR "education settings" OR "educational environments" OR "educational climates" OR "educational settings" OR "learning settings" OR "academic settings")) AND (TI("Health professions" OR "Health profession" OR "Health professional" OR "Health professionals" OR "Healthcare professions" OR "Healthcare profession" OR "Healthcare professional" OR "Healthcare professionals" OR "Health care professions" OR "Health care profession" OR "Health care professional" OR "Health care professionals" OR "Allied health" OR "Health science" OR "Health sciences" OR Biomed* OR dent* OR nurs* OR nutrition* OR diet* OR physiotherap* OR physio*therap* OR occupational therap* OR occupation therap* OR rad*therap* OR radiotherap* OR radiation therap* OR physical therap* OR veterinar* OR medical OR medicine OR audiolog* OR psych* OR “speech* path*” OR “speech* therap*” OR “speech and language path*” OR “speech-language path*” OR “language path*” OR “language therap*” OR “speech-language therap*” OR “speech-language therap*” OR midwi* OR physician*) OR AB("Health professions" OR "Health profession" OR "Health professional" OR "Health professionals" OR "Healthcare professions" OR "Healthcare profession" OR "Healthcare professional" OR "Healthcare professionals" OR "Health care professions" OR "Health care profession" OR "Health care professional" OR "Health care professionals" OR "Allied health" OR "Health science" OR "Health sciences" OR Biomed* OR dent* OR nurs* OR nutrition* OR diet* OR physiotherap* OR physio*therap* OR occupational therap* OR occupation therap* OR rad*therap* OR radiotherap* OR radiation therap* OR physical therap* OR veterinar* OR medical OR medicine OR psych* OR “speech* path*” OR “speech* therap*” OR “speech and language path*” OR “speech-language path*” OR “language path*” OR “language therap*” OR “speech-language therap*” OR “speech-language therap*” OR midwi* OR physician*)) AND (TI(school* OR college* OR universit* OR faculty OR "learning institute" OR "learning institutes" OR "learning institution" OR "learning institutions" OR "education institute" OR "education institutes" OR "education institution" OR "education institutions" OR "educational institute" OR "educational institutes" OR "educational institution" OR "educational institutions" OR "academic institute" OR "academic institutes" OR "academic institution" OR "academic institutions" OR education* OR curricul* OR mentor* OR precept* OR teach*) OR AB(school* OR college* OR universit* OR faculty OR "learning institute" OR "learning institutes" OR "learning institution" OR "learning institutions" OR "education institute" OR "education institutes" OR "education institution" OR "education institutions" OR "educational institute" OR "educational institutes" OR "educational institution" OR "educational institutions" OR "academic institute" OR "academic institutes" OR "academic institution" OR "academic institutions" OR education* OR curricul* OR mentor* OR precept* OR teach*)) AND (TI(tool* OR instrument* OR survey* OR quantitative* OR questionnaire* OR scale* OR inventor*) OR AB(tool* OR instrument* OR survey* OR quantitative* OR questionnaire* OR scale* OR inventor*)) AND (la.exact("ENG") AND PEER(yes)) AND (TI(opinion* OR attitude* OR view OR perspective* OR perceive* OR behaviour* OR percept* OR measur* OR assess* OR evaluat* OR views OR viewpoint OR viewpoints) OR AB(opinion* OR attitude* OR view OR perspective* OR perceive* OR behaviour* OR percept* OR measur* OR assess* OR evaluat* OR views OR viewpoint OR viewpoints)) | English  Peer-reviewed  Since 1940 | 1,957 |
| **ERIC** | ( TI("academic environment" OR "academic climate" OR "learning environment" OR "learning climate" OR "classroom environment" OR "classroom climate" OR "education environment" OR "education climate" OR "education setting" OR "educational environment" OR "educational climate" OR "educational setting" OR "learning setting" OR "academic setting" OR "academic environments" OR "academic climates" OR "learning environments" OR "learning climates" OR "classroom environments" OR "classroom climates" OR "education settings" OR "educational environments" OR "educational climates" OR "educational settings" OR "learning settings" OR "academic settings") OR AB("academic environment" OR "academic climate" OR "learning environment" OR "learning climate" OR "classroom environment" OR "classroom climate" OR "education environment" OR "education climate" OR "education setting" OR "educational environment" OR "educational climate" OR "educational setting" OR "learning setting" OR "academic setting" OR "academic environments" OR "academic climates" OR "learning environments" OR "learning climates" OR "classroom environments" OR "classroom climates" OR "education settings" OR "educational environments" OR "educational climates" OR "educational settings" OR "learning settings" OR "academic settings") ) AND ( TI("Health professions" OR "Health profession" OR "Health professional" OR "Health professionals" OR "Healthcare professions" OR "Healthcare profession" OR "Healthcare professional" OR "Healthcare professionals" OR "Health care professions" OR "Health care profession" OR "Health care professional" OR "Health care professionals" OR "Allied health" OR "Health science" OR "Health sciences" OR Biomed* OR dent* OR nurs* OR nutrition* OR diet* OR physiotherap* OR physio*therap* OR occupational therap* OR occupation therap* OR rad*therap* OR radiotherap* OR radiation therap* OR physical therap* OR veterinar* OR medical OR medicine OR pharmac* OR audiolog* OR psych* OR “speech* path*” OR “speech* therap*” OR “speech and language path*” OR “speech-language path*” OR “language path*” OR “language therap*” OR “speech-language therap*” OR “speech-language therap*” OR midwi* OR physician) OR AB("Health professions" OR "Health profession" OR "Health professional" OR "Health professionals" OR "Healthcare professions" OR "Healthcare profession" OR "Healthcare professional" OR "Healthcare professionals" OR "Health care professions" OR "Health care profession" OR "Health care professional" OR "Health care professionals" OR "Allied health" OR "Health science" OR "Health sciences" OR Biomed* OR dent* OR nurs* OR nutrition* OR diet* OR physiotherap* OR physio*therap* OR occupational therap* OR occupation therap* OR rad*therap* OR radiotherap* OR radiation therap* OR physical therap* OR veterinar* OR medical OR medicine OR pharmac* OR psych* OR “speech* path*” OR “speech* therap*” OR “speech and language path*” OR “speech-language path*” OR “language path*” OR “language therap*” OR “speech-language therap*” OR “speech-language therap*” OR midwi* OR physician) ) AND ( TI(school* OR college* OR universit* OR faculty OR "learning institute" OR "learning institutes" OR "learning institution" OR "learning institutions" OR "education institute" OR "education institutes" OR "education institution" OR "education institutions" OR "educational institute" OR "educational institutes" OR "educational institution" OR "educational institutions" OR "academic institute" OR "academic institutes" OR "academic institution" OR "academic institutions" OR education* OR curricul* OR mentor* OR precept* OR teach*) OR AB(school* OR college* OR universit* OR faculty OR "learning institute" OR "learning institutes" OR "learning institution" OR "learning institutions" OR "education institute" OR "education institutes" OR "education institution" OR "education institutions" OR "educational institute" OR "educational institutes" OR "educational institution" OR "educational institutions" OR "academic institute" OR "academic institutes" OR "academic institution" OR "academic institutions" OR education* OR curricul* OR mentor* OR precept* OR teach*) ) AND ( TI(tool* OR instrument* OR survey* OR quantitative* OR questionnaire* OR scale* OR inventor*) OR AB(tool* OR instrument* OR survey* OR quantitative* OR questionnaire* OR scale* OR inventor*) ) AND ( TI(opinion* OR attitude* OR view OR perspective* OR perceive* OR behaviour* OR percept* OR measur* OR assess* OR evaluat* OR views OR viewpoint OR viewpoints) OR AB(opinion* OR attitude* OR view OR perspective* OR perceive* OR behaviour* OR percept* OR measur* OR assess* OR evaluat* OR views OR viewpoint OR viewpoints) ) | English  Peer-reviewed  Since 1940 | 610 |
| **Cochrane library** | (("academic environment" OR "academic climate" OR "learning environment" OR "learning climate" OR "classroom environment" OR "classroom climate" OR "education environment" OR "education climate" OR "education setting" OR "educational environment" OR "educational climate" OR "educational setting" OR "learning setting" OR "academic setting" OR "academic environments" OR "academic climates" OR "learning environments" OR "learning climates" OR "classroom environments" OR "classroom climates" OR "education settings" OR "educational environments" OR "educational climates" OR "educational settings" OR "learning settings" OR "academic settings"):ti,ab,kw) AND ((MeSH descriptor: [Health Personnel] explode all trees) OR ("Health professions" OR "Health profession" OR "Health professional" OR "Health professionals" OR "Healthcare professions" OR "Healthcare profession" OR "Healthcare professional" OR "Healthcare professionals" OR "Health care professions" OR "Health care profession" OR "Health care professional" OR "Health care professionals" OR "Allied health" OR "Health science" OR "Health sciences" OR Biomed* OR dent* OR nurs* OR nutrition* OR diet* OR physiotherap* OR physio*therap* OR occupational therap* OR occupation therap* OR rad*therap* OR radiotherap* OR radiation therap* OR physical therap* OR veterinar* OR medical OR medicine):ti,ab,kw) AND ((MeSH descriptor: [Education] explode all trees) OR (school* OR college* OR universit* OR faculty OR "learning institute" OR "learning institutes" OR "learning institution" OR "learning institutions" OR "education institute" OR "education institutes" OR "education institution" OR "education institutions" OR "educational institute" OR "educational institutes" OR "educational institution" OR "educational institutions" OR "academic institute" OR "academic institutes" OR "academic institution" OR "academic institutions"):ti,ab,kw) AND ((tool* OR instrument* OR survey* OR quantitative* OR questionnaire* OR scale* OR inventor*):ti,ab,kw) AND ((opinion* OR attitude* OR view OR perspective* OR perceive* OR behaviour* OR percept* OR measur* OR assess* OR evaluat* OR views OR viewpoint OR viewpoints):ti,ab,kw) | --  Since 1940 | 214 |
